# *Enterobacter cloacae* Outbreak and Emergence of Quinolone Resistance Gene in Dutch Hospital

**DOI:** 10.3201/eid1205.050910

**Published:** 2006-05

**Authors:** Armand Paauw, Ad C. Fluit, Jan Verhoef, Maurine A. Leverstein-van Hall

**Affiliations:** * University Medical Center, Utrecht, the Netherlands

**Keywords:** qnrA, Enterobacter cloacae, integron, fluoroquinolones, plasmid-mediated, qnr, CTX-M-9, aminoglycoside, outbreak, research

## Abstract

An outbreak of *Enterobacter cloacae* infections with variable susceptibility to fluoroquinolones occurred in the University Medical Center Utrecht in the Netherlands in 2002. Our investigation showed that a *qnrA*1 gene was present in 78 (94%) of 83 outbreak isolates and that a *qnrA1*-encoding plasmid transferred to other strains of the same species and other species. The earliest isolate carrying this same plasmid was isolated in 1999. *qnrA1* was located in a complex integron consisting of the *intI1*, *aadB*, *qacEΔ1*, *sul1*, *orf513*, *qnrA1*, *ampR*, *qacEΔ1*, and *sul1* genes that were not described previously. On the same plasmid, 2 other class 1 integrons were present. One was a new integron associated with the *bla*_CTX-M-9_ extended-spectrum β-lactamase.

## Abstract 2

Plasmid-mediated *qnrA1* is an emerging resistance trait.

Multidrug-resistance among *Enterobacteriaceae*, including resistance to quinolones, is increasing. Although quinolone resistance is predominantly caused by chromosomal mutations, it may also result from a plasmid-encoded *qnr*-gene (1). The QnrA determinant, a 218–amino acid protein, protects DNA gyrase and topoisomerase IV from the inhibitory activity of quinolones (2). However, expression of *qnrA* alone is frequently insufficient to reach Clinical and Laboratory Standards Institute breakpoints for ciprofloxacin resistance. Since first identified in 1994 in the United States, *qnrA*-like genes have been sporadically identified in Enterobacteriaceae worldwide (3–9).

At the end of 2002, an outbreak of aminoglycoside-resistant *Enterobacter cloacae* infections with variable susceptibility for ciprofloxacin was detected in the University Medical Center Utrecht (UMCU), the Netherlands, involving >80 patients (10). The first aim of this study was to test the hypothesis that the variable susceptibility to ciprofloxacin of the outbreak strain was associated with plasmid-mediated *qnrA* and if so, to characterize the gene's molecular background and determine its ability to transfer in vitro as well as in vivo. Maximum circumstantial evidence for horizontal transfer in vivo with the outbreak strain as donor would be obtained if the following observations were made: 1) different species or strains collected from the same patient harbored the same *qnrA*-encoding plasmid; 2) this same *qnrA*-encoding plasmid was not found in patients without an epidemiologic link to the outbreak. The second aim of this study was to determine to what extent the *qnrA* gene is an emerging resistance problem in our hospital.

## Materials and Methods

### Bacterial Isolates

A total of 1,167 isolates were tested for a *qnrA* gene. Group I consisted of 178 *E. cloacae* pulsed-field gel electrophoresis (PFGE) typed isolates obtained from January 2001 to August 2003 from 159 patients (10). Of these, 83 tobramycin-resistant isolates obtained from 83 patients belonged to 1 clonal lineage (cluster I, outbreak strain). Five of these patients also carried a tobramycin-susceptible variant of the clonal lineage (I^A^). The remaining 95 *E. cloacae* isolates contained 5 small clusters of 2 isolates each (III–VII), 1 cluster with 6 isolates (VIII), 1 cluster with 3 isolates (II), and 70 unique strains.

Groups II and III consisted of aminoglycoside-resistant, gram-negative bacteria identified in the hospital database that were other than the outbreak strain; these bacteria were isolated from patients with an outbreak strain (group II) as well as from patients not involved in the outbreak but admitted in the same period (January 2001–August 2003) (group III). Aminoglycoside resistance was the selection criterion because the outbreak strain was aminoglycoside-resistant, and these isolates are stored routinely in our laboratory. Group IV consisted of 867 *Enterobacteriaceae* isolates comprising 8 different species collected from 3 different origins: 269 clinical isolates from UMCU (1994–2000), 514 isolates from 23 European hospitals (1997–1998), and 84 fecal screening isolates from 53 patients at admission at UMCU (2000) (11).

### Identification and Susceptibility Testing

Identification and susceptibility testing of isolates obtained through 2000 were performed by using the VITEK1 System with AMS R09.1 software (bioMérieux, Marcy-L'etoile, France); isolates obtained after 2000 were tested by using the Phoenix 100 Automated Microbiology System version V3.22 software (Becton Dickinson Biosciences, Sparks, MD, USA). For susceptibility testing, Clinical and Laboratory Standards Institute guidelines were used (12). In the conjugation experiments, MICs were determined by using Etest (AB Biodisk, Solna, Sweden).

### Genotyping and Characterization of β-Lactamases

*E. cloacae* isolates were typed by PFGE. *Citrobacter freundii*, *Escherichia coli*, and *Klebsiella pneumoniae* were typed by PFGE and random amplified polymorphic DNA (13). To determine the kind of β-lactamases the outbreak strain expressed, isoelectric focusing (IEF) was performed with Phastgels (pH gradient 3–9) with the PhastSystem (Pharmacia AB, Uppsala, Sweden) (14). β-lactamases of isoelectric pH (pI) 5.6 (TEM-1), pI 7.6 (SHV-2A), and pI 8.2 (*bla*_CTX-M-9_) and a broad range pI calibration set (Amersham Biosciences, Little Chalfont, UK) were used. β-lactamases were detected with nitrocefin (Oxoid, Basingstoke, UK).

### Detecting and Characterizing Resistance Genes

Target DNA for polymerase chain reaction (PCR) assays was extracted by heating bacterial suspensions for 10 min at 95°C. *qnrA*, *bla*_CTX-M_, and *aadB* were detected by PCR with primers and annealing temperatures described in the Table. The outbreak strain carried an integron containing an *aadB* gene encoding aminoglycoside resistance (17). Primers were developed to detect *aadB* gene and the downstream 3´-conserved segment (CS) of the integron in the same PCR (*aadB*-3´CS). PCR assays were performed for 30 or 35 cycles. The AmpC PCR tests were performed as described earlier, except that a single PCR format was used (18).

The *bla*_CTX-M_ gene from *E. cloacae* 02-477 was sequenced by using CTX-M-9 group sequence primers (Table). The flanking regions of the *qnrA* gene and the *bla*_CTX-M-9_ gene were determined by using a PCR and DNA sequencing strategy based on the sequences from In7, In36, In37, In60, and an integron from *E. coli* O159 (5,9,19–22). To confirm that the gene cassettes were part of a complex integron with *qnrA* or *bla*_CTX-M-9_, we used the Expand Long Template PCR system (Roche, Woerden, the Netherlands) employed primers to amplify sequences between the *qnrA* or *bla*_CTX-M-9_ and the possible gene cassettes. All amplified products were (partly) sequenced for confirmation. Sequencing was performed with Qiagen Quick (Qiagen, Westburg b.v., Leusden, the Netherlands) purified PCR products by using the BigDye Terminator v1.1 Cycle Sequencing Ready Reaction Kit and a 3100 capillary DNA sequencer (Applied Biosystems, Nieuwerkerk a/d Yssel, the Netherlands).

**Table:** Oligonucleotides used for polymerase chain reaction amplification and sequencing

Target	Primer	5´–3´ sequences	GenBank accession no.	Nucleotide positions	Annealing temperature (°C)	Amplicon size (bp)	Source
*QnrA*	qnrAR	AGG AAG CGC CGC TGA GAT TG	AY070235	762–743	56	281	This study
	qnrAF	CTA TGC CGA TCT GCG CGA TG	AY070235	482–501			This study
*aadB-3´CS*	aadB	TGG AGG AGT TGG ACT AT	AY173047	251–267	55	432	This study
	3´CS	AAG CAG ACT TGA CCT GA	M73819	1342–1326			(15)
*bla*_CTX-M-_: most	ctx-m-uni-F	CGA TGT GCA GTA CCA GTA A	U95364	214–232	50	538	This study
	ctx-m-uni-R	ATA TCG TTG GTG GTG CC	U95364	751–735			This study
*bla*_CTX-M-_: 2,4,5,6,7,20,Toho-1	ctx-m-2F	ATG ATG ACT CAG AGC ATT CG	X92507	6–25	58	884	(16)
	ctx-m-2R	TTA TTG CAT CAG AAA CCG TG	X92507	889–870			(16)
*bla*_CTX-M-_: 3,10,11,12,15,22,25	ctx-m-10-1F	ATG GTT AAA AAA TCA CTG CG	X92506	63–82	60	872	This study
	ctx-m-10-4R	AAA CCG TTG GTG ACG AT	X92506	934–918			This study
*bla*_CTX-M-_: 9,13,14,15,16,17,18,19,24,Toho-2 and –3	ctx-m-9F	AGA CGA GTG CGG TGC AGC AA	AJ416345	217–236	67	773	This study
	ctx-m-9R	GAT TCT CGC CGC TGA AGC CA	AJ416345	989–970			This study
Sequence *bla*_CTX-M-9_ group	ctx-m-9-1F	TGG TGA CAA AGA GAG TGC AAC G	AJ416345	133–154			This study
	ctx-m-9-MF	GGA GGC GTG ACG GCT TTT	AJ416345	576–593			This study
	ctx-m-9-MR	AAA AGC CGT CAC GCC TCC	AJ416345	593–576			This study
	ctx-m-9-4R	TCA CAG CCC TTC GGC GAT	AJ416345	1007–990			This study

### Conjugation Experiments

For conjugation experiments, an *E. coli* K12 and a tobramycin-susceptible clinical *E. cloacae* (03-702) isolate of PFGE cluster I^A^ were used as recipients. An *E. cloacae* (02-477) belonging to PFGE cluster I was used as donor. Conjugation was performed as described (23). MacConkey agar plates containing tobramycin (8 μg/mL) were used for counter selection, and transconjugants were selected on colony form. Conjugation was confirmed by a *qnrA*-specific PCR. Secondly, transconjugant *E. coli* C02-477A was used as a donor for *qnrA*-negative *E. cloacae* 03-702 belonging to cluster I^A^. Transconjugants were selected by using 15 μg/mL ampicillin-clavulanic acid and 5 μg/mL tobramycin. Transconjugants were characterized as described above.

### Detecting Resistance Genes on Plasmid by Southern Hybridization

Plasmids were isolated with the Qiagen Plasmid Maxi Kit (Qiagen). Plasmid DNA was separated on 1% PFGE agarose (Bio-Rad Laboratories, Richmond, CA , USA) in 0.5× Tris-borate-EDTA, 0.05 mmol/L thiourea buffer at 14°C in CHEF DR-II apparatus (Bio-Rad). Run time was 22 h with a voltage of 6 V/cm and a linearly ramped pulse time of 30 to 70 s. The DNA was blotted and hybridized. The probes were PCR amplification products obtained with primers used to detect *aadB*-3´-CS, *bla*_CTX-M-9_, and *qnrA* genes (Table). Products were labeled with the AlkPhosDirect Reaction Kit (Amersham Biosciences) and detected with CPD-Star (Amersham Biosciences).

## Results

### *qnrA1* in Outbreak Strain

For 78 (94%) of the 83 *E. cloacae* isolates in cluster I (outbreak strain), the *qnrA*-specific PCR was positive. To confirm results from the PCR, 2 fragments were sequenced. The obtained sequences were identical to the published sequence of *qnrA1* (GenBank accession no. AY070235).

Susceptibility testing showed that 87% of the 83 outbreak isolates were resistant or intermediate resistant to ciprofloxacin (43% resistant, 43% intermediate resistant), 100% were resistant to tobramycin, 63% to gentamicin, 2% to amikacin, 100% to ceftriaxone, 12% to trimethoprim-sulfamethoxazole, and 0% to carbapenems. A total of 81 (98%) of the 83 isolates harbored an *aadB* containing integron.

IEF showed the presence of a β-lactamase with a pI of ≈8.2, which suggested the presence of either an AmpC β-lactamase or a CTX-M type extended-spectrum β-lactamase. No AmpC-specific amplification products were obtained. Eighty-two (99%) of the 83 isolates harbored a *bla*_CTX-M_ gene. DNA sequencing showed the presence of *bla*_CTX-M-9_.

**Figure 1:**
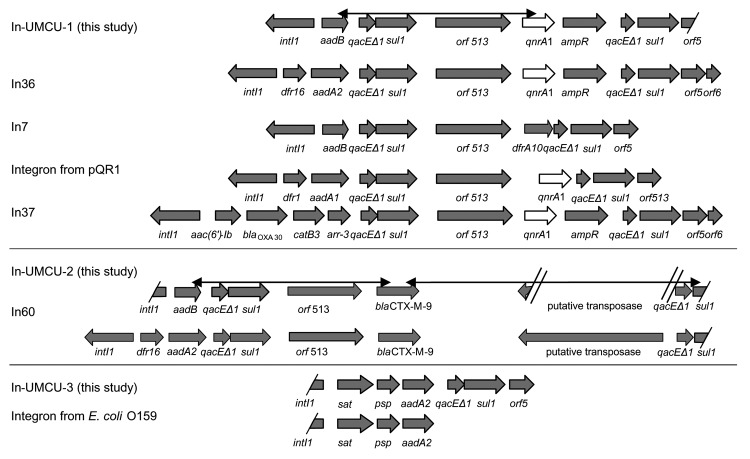
Schematic presentation of integrons on pQC compared with previously described integrons (5,9,19,21,22). The black double-pointed arrows indicate the product amplified with an Expand Long Template PCR system (Roche, Woerden, the Netherlands), demonstrating a link between the *qnrA* and *bla*_CTX-M-9_ genes and their respective integrons.

The plasmid (pQC) of conjugant *E. coli* C02-477A was isolated, and its size was estimated at 180 kb by agarose gel electrophoresis. Southern blotting that used specific probes confirmed that pQC contained the *qnrA1* gene, the *bla*_CTX-M-9_ gene, and the integron with an *aadB* gene cassette (data not shown). Sequences flanking the *qnrA1* and *bla*_CTX-M-9_ genes were comparable with 3 previously described class 1 integrons (Figure 1). The first integron (In-UMCU-1 accession no. AY987395), containing the *qnrA1* gene, had the same additional structures as In36, *orf513*, *qnrA1*, *ampR*, plus a second copy of the 3´-conserved segment. The In36 integron contained the gene cassettes *drf16* and *aadA2*, while In-UMCU-1 contained only the *aadB* gene cassette. In addition, the DNA sequences between the second s*ul1* gene and *orf5* (bp 9606–9624 of In36) differed from the sequence of In-UMCU-1 (5). The second integron (In-UMCU-2, accession no. DQ108615), which contained *bla*_CTX-M-9_, was comparable to In60, but In60 contained the *drf16* and *aadA2* gene cassettes, while In-UMCU-2 contained the *aadB* gene cassette (21). The third integron (In-UMCU-3, accession no. DQ019420), which contained the gene cassettes *sat*, *psp*, and *aadA2*, was described previously in an enterotoxigenic *E. coli* O159 isolated in Japan (22). PCR amplification of the *aadA2* gene of the donor, recipient, and transconjugants indicated that this third integron was also located on pQC.

### Evidence for Transfer of *qnrA* in vitro

**Figure 2:**
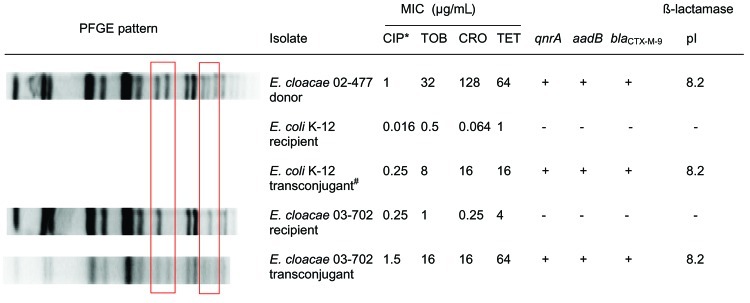
Pulsed-field gel electrophoresis (PFGE) patterns, susceptibility patterns, and key resistance genes for recipient and transconjugants in in vitro conjugation experiments. Boxes denote the area of variability in the PFGE patterns between isolates with and without pQC. CIP, ciprofloxacin; TOB, tobramycin; CRO, ceftriaxone; TET, tetracycline. *Escherichia coli transformant served as donor for *Enterobacter cloacae* 02-0702.

In vitro conjugation experiments showed that pQC could be transferred both from and to the outbreak strain (Figure 2). pQC was successfully transferred from *E. cloacae* 02-477 to recipient *E. coli* K12. The resulting transconjugant *E. coli* was subsequently used as donor to transfer pQC to a type I^A^ *E. cloacae* (03-702), which resulted in a successful transfer of pQC. pQC conferred increased ciprofloxacin MICs (from 6- to 10-fold) and resistance to tobramycin, tetracycline, and ceftriaxone to the transconjugants (Figure 2). Acquisition and loss of pQC were associated with 2 changes in the PFGE pattern.

### Evidence for Transfer of *qnrA1* in vivo

Different species or strains collected from the same patient harbored the same pQC. From 22 of the 53 patients with an outbreak strain, 35 other tobramycin-resistant, gram-negative clinical isolates were available. Eleven different strains obtained from 11 patients were positive for *qnrA1*, *bla*_CTX-M-9_, and *aadB*-3´-CS. These comprised 4 different species: *C. freundii* (n = 1), *Enterobacter aerogenes* (n = 1), *E. coli* (n = 7), and *K. pneumoniae* (n = 2). Plasmid isolation from 6 *E. coli* and 1 *K. pneumoniae* yielded a plasmid of the same size as the pQC in the outbreak strain. Because of its large size and possibly a very low copy number, only small amounts of plasmid DNA could be isolated. These amounts were insufficient to perform further comparative analyses by restriction fragment analysis or Southern blotting.

Some *E. cloacae* strains with a strong epidemiologic link to the outbreak strain were also pQC positive. All isolates belonging to clusters III, VII, and VIII contained pQC as well as 5 *E. cloacae* isolates with a unique genotype. Plasmid isolation of 3 strains again showed a plasmid of the same size as the outbreak pQC. Three of the 5 unique isolates were obtained from patients who also harbored the outbreak strain.

**Figure 3:**
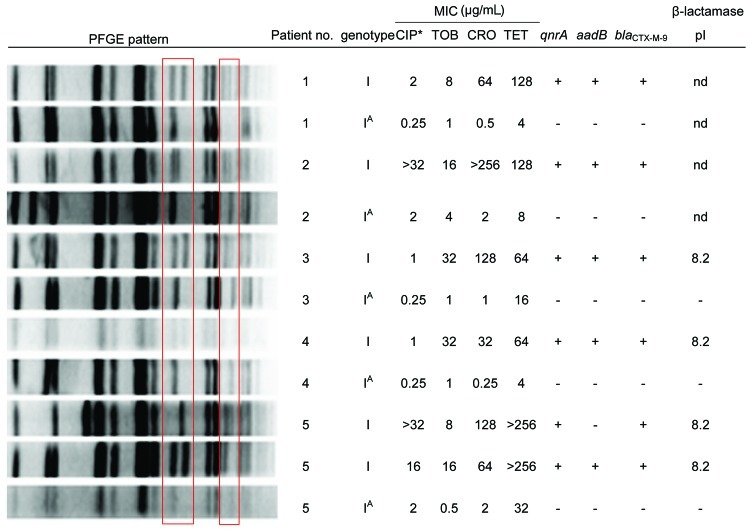
Pulsed-field gel electrophoresis (PFGE) patterns, susceptibility patterns, and key resistance genes of *Enterobacter cloacae* isolates from patients harboring isolates belonging to genotype I as well as I^A^. Boxes denote the area of variability in the PFGE patterns between isolates with and without pQC. CIP, ciprofloxacin; TOB, tobramycin; CRO, ceftriaxone; TET, tetracycline.

The *qnrA* gene, the *aadB*-containing integron, and the *bla*_CTX-M-9_ could not be detected in PFGE cluster I^A^, which is closely related to the outbreak strain (Figure 3). The loss of these genes was associated with increased susceptibility to ciprofloxacin, tobramycin, ceftriaxone, and tetracycline. In addition, an identical change in the PFGE pattern was observed, as in the in vitro experiments. These results suggest that the host may lose pQC in vivo.

### *qnrA1* Recent Emergence as Clinical Problem

pQC was not found in isolates obtained from patients without an epidemiologic link to the outbreak. No *qnrA1* gene was detected in any of 83 aminoglycoside-resistant gram-negative organisms (44 *E. coli*, 19 *K. pneumoniae*, 4 *Proteus mirabilis*, 2 *Klebsiella oxytoca*, 2 *E. cloacae*, 1 *Enterobacter* sp., 7 *C. freundii*, 4 *Serratia marcescens*) obtained from 74 patients admitted to wards not involved in the outbreak during the outbreak period. Neither was *qnrA*1 detected in any of the 269 UMCU isolates or the 84 community isolates.

Only 1 *qnrA1*-positive isolate was found in the 514 European isolates. This *qnrA1*-positive isolate was an *E. cloacae* organism isolated in 1999 at a surgical ward at UMCU that belonged to cluster III. The other 2 cluster III isolates were isolated at the same surgical ward during the outbreak period.

## Discussion

We report a nosocomial outbreak with an R-plasmid–encoded *qnrA1* gene. This plasmid (pQC) was first detected in an *E. cloacae* isolated in 1999 and subsequently in another *E. cloacae* strain that caused a large outbreak in our hospital, starting in 2001. Strong evidence is provided that this outbreak strain was the source from which pQC disseminated to other strains of the same species and other species by horizontal gene transfer. The *qnrA1* gene was not detected in any of the hospital isolates (1994–2003) tested without an epidemiologic link to the outbreak strain, indicating that *qnrA1* is a new emerging resistance trait in our hospital.

pQC contained 3 different class 1 integrons. One integron was identical to an integron detected in an *E. coli* O159 from Japan (22). The 2 other integrons were complex integrons, In-UMCU-1 and In-UMCU-2, which were not described previously. Complex integrons are composed of a 5´-CS, gene cassettes, 3´-CS, *qacΔE*, *sulI*, additional genes, *qacΔE*, and *sulI*. These additional genes differ from gene cassettes by lacking a 59-bp element and having their own promoter (24). The *qnrA1* gene in In-UMCU-1 was also present as an additional gene, as was the case for the 3 previous characterized *qnrA1* genes in In36, In37, and the complex integron of pQR1 (5,9). The sequences of these genes were identical for In-UMCU-1, In 36, and In37, and slightly different for the integron on pQR1. The gene cassette content of the 4 integrons, however, was different, although all 4 possessed a gene encoding aminoglycoside resistance. All *qnrA1*-positive isolates reported in the literature also show resistance to cephalosporins (1,4–9,25–27). Therefore, *qnrA1* seems to be closely associated with resistance to cephalosporins and aminoglycosides. How these comparable but different complex integrons arose is unclear. Either the same additional genes became associated with different integrons or the gene cassettes in an original complex integron were exchanged.

Our study confirmed previous findings that the presence of *qnrA1* does not necessarily lead to MICs above Clinical and Laboratory Standards Institute breakpoints for resistance to ciprofloxacin (1,3,7,25). Therefore, the presence of *qnrA1* had no therapeutic consequences for the patients from whom these isolates were obtained. However, the increased MIC may provide the host bacterium a selective advantage in an environment of low concentrations of quinolones, increasing the bacterial numbers and therefore the absolute chance of a chromosomal mutation encoding resistance (7,25). The presence of a *qnrA*-carrying plasmid might even enhance the mutation rate encoding quinolone resistance (1). Furthermore, acquisition of *qnrA* by a host bacterium that already contains quinolone resistance mechanisms may raise MICs above the LCSI breakpoints (25,28,29). As shown in this study, the same plasmid may cause fluctuation in susceptibility in MICs in different recipients because of variation in porin expression or mutations in the gyrase or efflux pump-encoding genes (2).

In conclusion, in a hospital setting the *qnrA* gene is advantageous for the host bacterium. Because of this gene's location on promiscuous R-plasmids, it is likely to emerge worldwide.
